# The effect of patients’ feedback on treatment outcome in a child and adolescent psychiatric sample: a randomized controlled trial

**DOI:** 10.1007/s00787-018-1247-4

**Published:** 2018-11-03

**Authors:** Rint K. de Jong, Heddeke Snoek, Wouter G. Staal, Helen Klip

**Affiliations:** 1 Karakter Child and Adolescent Psychiatry, Dr. E. Schattenkerkweg 1, 8025 BW Zwolle, The Netherlands; 2 Leiden Institute for Brain and Cognition, Leiden, The Netherlands; 3 Karakter Child and Adolescent Psychiatry University Centre, Reinier Postlaan 12, 6525 GC Nijmegen, The Netherlands; 4 Radboud University Medical Centre, Department of Psychiatry, Nijmegen, The Netherlands

**Keywords:** Feedback informed treatment, Randomized controlled trial, Child and adolescent psychiatry, Autism spectrum disorder, Quality of Life, Symptom severity

## Abstract

The systematic use of feedback from patients on treatment progress and treatment satisfaction is a promising method to increase treatment effectiveness. The extent to which this also applies to the treatment of children with severe psychiatric problems is not clear. We conducted a Randomized Controlled Trial (RCT) to study the effect of adding Feedback Informed Treatment (FIT) to care as usual in a child psychiatric sample. Quality of Life (QoL) was used as the primary outcome measure and symptom severity as the second. Fifty-one therapists from eight Autism Care Teams in a multi-center facility for Child and Adolescent Psychiatry (Karakter) participated and were cluster randomized to the FIT condition (*n* = 4 teams) or the Care as Usual (CAU) condition (*n* = 4 teams). Children aged 6–18 years, mainly with an Autism Spectrum Disorder (ASD) and treated in one of the Autism Care Teams were allocated to the FIT condition (*n* = 86) or the CAU condition (*n* = 80). Results indicated that adding FIT leads to an increased QoL [*F* (2,165) = 3.16, *p *= 0.045]. No additional effects were observed for symptom severity decrease [*F* (2,158) = 0.19, *p *= 0.825]. No interaction with time was found for QoL nor symptom severity. Adding FIT in a child psychiatric setting may increase QoL, but does not appear to decrease symptom severity as compared with CAU. It is suggested that FIT positively changes parents’ expectations. Results should be replicated in other child psychiatric samples and with an extended theoretical model.

## Introduction

The systematic use of patients’ feedback is a promising method to increase the effectiveness of treatment [1–7]. Feedback provides critical information on treatment progress. Furthermore, adding feedback about treatment satisfaction may strengthen the therapeutic alliance between patient and therapist, which is known to be one of the most important contributors to treatment effect [8–12]. Systematic feedback stimulates reflection on treatment effects in relation to the quality of the therapeutic alliance experienced. In particular, it reflects the contribution of the therapist as an important partner in this alliance. It is in line with current discourse regarding the emancipation of the patient’s role in healthcare, e.g., a trend towards shared decision-making about treatment [13, 14].

Miller and others developed “Feedback Informed Treatment” (FIT): a systematic way to incorporate feedback in each treatment session [15]. FIT stimulates a culture of feedback within treatment. The therapist inquires about the patient’s well-being at the start of every session and the patients’ experience of the session at the end of the session. The therapist and the patient reflect in a deliberate way on this feedback using an immediate visualization of the feedback in a graph. Therapist and patient are able to adapt their goals, approach, method and frequency based on the feedback and the reference data shown in the graph. Together they can generate valuable information about their alliance, which enables them to become attuned to each other in a more sophisticated way. The addition of systematic feedback can be an important instrument in providing children with severe problems with all the value of treatment more effectively. In this way, the use of patients’ feedback can also be an important communication tool in the context of personalized care.

The effect of feedback on treatment outcome, in general, has been the subject of research for more than two decades. Most of the studies that were published reported small–moderate positive effects on different outcome measures [1–7, 16]. These studies also show several moderating factors for the effect of feedback. In general, more enhanced effects are seen in patients who are at risk of treatment failure [6, 17]. Therapist characteristics also moderate the effect of feedback, as shown in studies by Lutz et al. [18] and De Jong et al. [19]. It is under debate what kind of feedback should ideally be given and to whom. It is suggested that feedback given to both therapists and patients is more effective than feedback to therapists alone [2, 6, 19, 20]. It is also thought that the effect of feedback is elevated by applying a formalized structure for using feedback [16] and by a clinical decision tool that is based on feedback measures and expected treatment response [6, 21, 22]. The research questions become more specific around the key question: when and for whom is feedback effective? [1, 5].

In 2016, the Cochrane Collaboration published a comprehensive systematic review of feedback studies in adults [23]. At that time, the conclusion was that insufficient evidence was available to demonstrate the efficacy of formalized feedback in the treatment of common mental health disorders. It was suggested that most of the findings in studies were at high risk of different types of bias and relatively low quality with respect to evidence. It was suggested that future research should take into account specific groups, such as children, also with a clearly defined assessment of symptom severity based on standard classification systems and using multiple and additional outcome measures, not assessed by the therapist involved in the study [1–3, 23].

Previous research on feedback in treatment has mainly been conducted in the field of adult healthcare and the care for people with relatively mild concerns. The extent to which these findings can be replicated for people with more severe problems has been questioned, and whether treatment effects can last over a longer period. The latter emphasizes the need for longitudinal studies [1–3, 17]. Research on this topic in more specific areas, such as child mental healthcare, has been recommended in recent systematic reviews [1–3].

Research involving the FIT method or other feedback systems is rare in children and adolescents. A cluster randomized study showed that young people aged 11–18 improved faster when clinicians received weekly feedback about treatment progress [24]. Another pilot study, administered by parents, showed that an increase in conversation about treatment progress had a positive effect on the child’s functioning and the therapeutic relationship [25].

Most of the feedback-related studies have used symptom severity as an outcome measure, using checklists such as the Outcome Questionnaire 45 (OQ-45) and Symptom Checklist 90 (SCL-90); See [1, 2] for an overview. In the past decade, a paradigm shift has occurred in the criteria used to evaluate (positive) health as a treatment outcome. The definition of health has been extended to the patients’ experience of their health state. This patient-oriented outcome is covered in the concept of (health-related) Quality of Life (QoL) [26, 27]. However, only a few feedback effect studies have used QoL as an outcome measure. A cluster randomized controlled trial showed that an intervention structuring the patient–clinician dialog (feedback) to focus on patients’ views positively influenced QoL in adult patients with schizophrenia [28]. In another randomized controlled trial, the quarterly routine use of outcome measures did not improve subjective outcomes (like QoL) in adult mental health services [29]. Kendrik et al. recommended collecting additional outcome measures such as the QoL [23].

Given these recommendations and the limited literature on the use of FIT in child and adolescent psychiatric settings, we conducted a cluster randomized trial in a large sample of patients, aged under 18 years. Our objective in this FIT trial was to evaluate whether FIT increased QoL and decreased symptom severity compared with children and adolescents who did not use FIT in their treatment.

## Methods

### Trial design

A cluster randomized controlled trial (RCT) was conducted between June 2014 and December 2016. This cluster randomized design was used to minimize contamination bias within locations and to stimulate therapists in the experimental group to encourage each other to use FIT (see also Kendrik 2016 [23]). Clusters were the outpatient Autism Care Teams of eight different locations of *Karakter*, our center for child and adolescent psychiatry in the Netherlands. These were randomly allocated to the experimental condition (4 teams: Almelo, Arnhem, Ede, Tiel) or the control condition (4 teams: Apeldoorn, Enschede, Nijmegen, Zwolle). All teams used the same clinical protocols.

The Medical Ethics Committee for Arnhem–Nijmegen issued a positive judgement for this study (NL number 48681.091.14 METC no. 2014/144). This trial was registered with the ISRCTN Clinical Trial Registry with trial registration number ISRCTN12284149 (10.1186/ISRCTN12284149).

### Participants

Patients eligible for participation in the study were recruited between June 2014 and June 2015 from all patients referred by primary, secondary and tertiary Health Care services to one of the eight Autism Care Teams for diagnostics and treatment. Eligibility criteria were as follows: (1) aged between 6 and 18 years, (2) referred to one of the eight participating Autism Care Teams, (3) Dutch speaking, (4) ability to complete the outcome questionnaires digitally, and (5) Informed Consent was given by the parents and the child if 12 years or older. Patients were excluded if (1) they did not receive any treatment after diagnostics; (2) treatment was given in another team or by a therapist who was not trained in FIT; (3) FIT was used in fewer than three sessions or (4) the parents’ response to the repeated outcome assessment was less than two. Diagnosis and classification according to the Diagnostic and Statistical Manual for mental disorders (DSM-IV) [30] were confirmed by a psychiatrist for all children. This was based on a multidisciplinary assessment including a psychiatric observation, developmental assessment, parental interview and school questionnaires. Treatment plans were outlined by a multidisciplinary team and confirmed by the psychiatrist based on the diagnosis and in accordance with treatment protocols. All children were able to communicate verbally with their therapist. All children who met these criteria and started treatment in one of the Autism Care Teams were eligible for participation.

Parents or caregivers of all children, referred to one of the eight Autism Care Teams between June 2014 and June 2015, were informed by letter and orally about the goal and design of this study and were asked to agree to participate by signing an Informed Consent form. All children aged 12 years and older were also asked to give their Informed Consent.

**Fig. 1:**
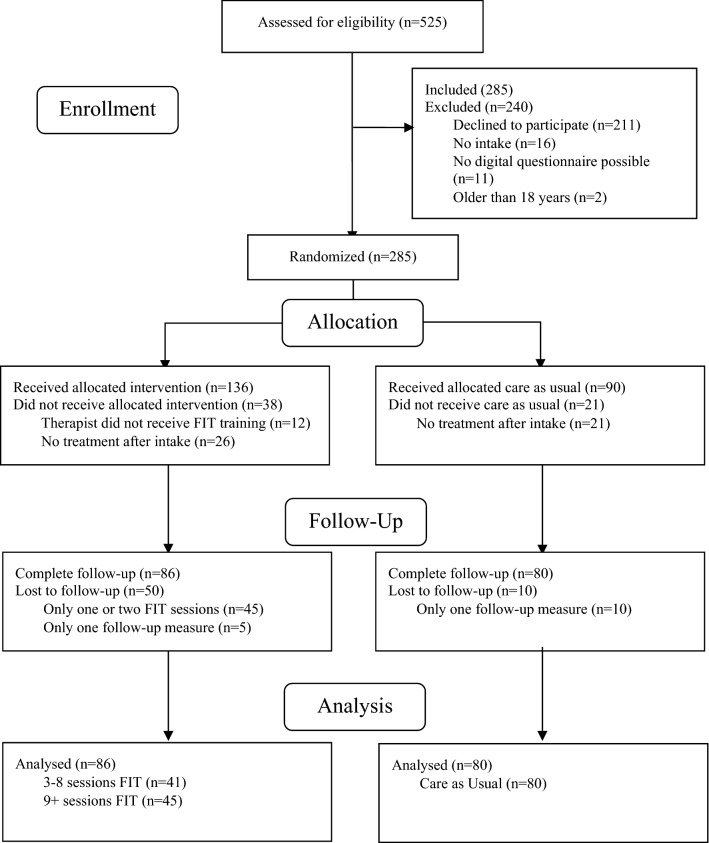
Flowchart of this study

The flow diagram of the study is presented in Fig. 1. A total of 525 children were assessed for eligibility. Of those, we had to exclude 240 children because they did not meet the inclusion criteria. All 285 eligible participants were allocated to the Experimental condition (FIT group, *n* = 174 participants) or the Control condition (CAU group, *n* = 111 participants), depending on the location to which they were referred. In the FIT group, we excluded another 26 participants who did not receive treatment after diagnostics, 12 participants who received treatment from a non-FIT-trained therapist, 45 participants who were asked for feedback fewer than three times and 5 participants with no repeated outcome measurement. Therefore, 86 patients were included in the FIT group for analyses. In the CAU group, 21 participants did not receive treatment after diagnostics, and 10 participants did not have repeated outcome measures. Therefore, 80 participants were included in the CAU group for analyses. Based on patient-focused feedback theories which suggest that feedback is more effective when given immediately, frequently and systematically, we expected the number of sessions with FIT to moderate the effect on treatment [4, 31]. Therefore, we divided the FIT group into a group with 3–8 sessions (*n* = 41) and a group with 9 or more session (*n* = 45).

### Therapists

Fifty-one therapists associated with one of the multidisciplinary Autism Care Teams were included in this study, including psychiatrists, psychotherapists (Cognitive Behavioral Therapy and System Therapy), psychologists, social workers and family workers. No differences were found in the multidisciplinary makeup of the teams or the mean age, years of experience and gender. Therapists in the experimental FIT group attended a one-day training course in the principles and use of FIT by a certified trainer in accordance with manual two of the manuals published by the International Center for Clinical Excellence (ICCE) [32].

Nine therapists left the teams during the study because of organizational reorganization and were replaced by new therapists. Another sixteen new therapists joined the teams. In the case of the FIT group, all new therapists were trained in half a day. Therapists in the FIT group attended monthly team supervision about FIT during the first 12 months and twice monthly for another 6 months. This was undertaken with a trained supervisor. Two research assistants regularly checked if therapists added new FIT data per patient in the digital fit-outcomes program.

### Interventions

Therapists in the experimental group added the use of feedback in their treatment sessions with the children and parents who participated in this study. They were also given the option to use it voluntarily with other patients not participating in this study. To standardize the use of feedback, therapists used FIT as described in manual two of the ICCE Manuals on Feedback Informed Treatment [32] and the additional web-based program *fit*-*outcomes* (http://www.fit-outcomes.com). At the start of each session, the child and the parents (if attending the session) completed the Dutch translation of the Outcome Rating Scale (ORS) about the child on an iPad by putting a mark on a visual ten-centimeter scale, ranging from ‘very bad’ on the left side to ‘very good’ on the right side. The ORS consists of four items about the well-being of the child [at (1) individual level, (2) family level (3) social level, (4) general level]. Completing the ORS took 2 minutes. The Total ORS score (range 0–40) was immediately shown in a graph, which reflected the progress of the patient over the treatment period. This progress was set against two reference lines that indicated the average course of successful and non-successful treatment outcomes respectively, given the initial patient ORS score based on an extensive database. Similarly, at the end of each session, the child and the parents (if attending the session) completed the Dutch translation of the Session Rating Scale (SRS) for themselves, which also contains four items about the way they perceived the session [(1) relationship (2) goals and setting, (3) approach and methods, (4) overall]. While the ORS was completed for the child in all cases, the SRS was filled in for the session participant, which could be the child or the parents. The Total SRS score (range 0–40) was shown in the same graph and reflected the curve of the way the patient was experiencing treatment. The SRS curve was also set against a cut-off line based on the same large dataset. The therapist and the patient were encouraged to discuss the results shown in the graph. This conversation is the main point of FIT and creates added value.

The FIT approach meets the criteria for Evidence-Based Practice of the American Psychological Association [33] as recognized by the Substance Abuse and Mental Health Services Administration (see also Tilsen [34]). Research has shown that the translated Dutch ORS and SRS have sufficient reliability and a limited validity for the Dutch population [35, 36]. Janse et al. concluded that the Dutch ORS and SRS were suitable questionnaires for following progress during treatment, but recommended the use of a second questionnaire for measuring treatment outcome [36]. So far, only one study has reported the psychometric properties of the ORS for children. This demonstrated that the instrument had sufficient validity and good reliability [37].

The care provided in the experimental group as well as in the control group was in accordance with the same clinical protocols. The vast majority of children received child psycho-education (generally once a week, over a 10-week period). This was followed in some instances by a (social) skills training (weekly, over a period of 10 weeks), emotion or behavior regulation skills training, Psycho-motoric Therapy (generally once a week, over a period of 15 weeks), (Cognitive) Behavioral Therapy during 15 weeks and pharmacotherapy. Parents attended psycho-education and parent mediation therapy ranging from low-frequency sessions with a psychologist (for example, once per month) to intensive parental training set-up in their home environment (for example twice a week, for 25 weeks) by a family worker.

### Measures

Behavioral problems at baseline were measured using parents’ ratings on the Dutch translation of the Child Behavior Checklist (CBCL) at the start. This is a widely used standardized questionnaire for children aged 6–18 years [38]. The CBCL is a parents’ rating scale, which measures children’s general problem behavior and internalizing and externalizing behavior, while more specific problem behaviors are assessed with supplementary scales. To compare the experimental and control groups, we used the total scale, externalizing behavior scale, internalizing behavior scale and the subscales [38]. Internal consistencies (Cronbach’s Alpha) for the Dutch version were found to be > 0.90 [39].

To reduce observer rating bias, we decided to use two outcome measures which (1) differ from the feedback measures collected by the therapist during the treatment (see also Shimokawa 2010 [6]) and (2) are also assessed by other people (in this case the parents) than the therapists (see also Kendrick 2016 [23]).

Assessing the treatment outcome in terms of QoL, we used the Dutch translation of the Kidscreen 27 Questionnaire [40, 41]. The Kidscreen 27 has 27 items representing five dimensions: Physical Well-being, Psychological Well-being, Autonomy and Parents, Peers and Social Support, and School Environment. Completing the Kidscreen 27 takes 10–15 min. The items are scored on a five-point Likert scale, with a range from one (never) to five (always). The item scores are summarized per dimension and transformed into a T-score and percentiles. The intern consistency of the dimensions ranges from above mean to good (Cronbach’s Alpha > 0.70) [42].

To assess treatment outcome in terms of change in symptom severity level, we used the Dutch translation of the Youth Outcome Questionnaire (Y-OQ30) [43–45]. The Y-OQ 30 has 30 items and can be completed in 10–15 min using a five-point Likert scale with a range from 0 (never) to 4 (always). The Y-OQ30 has six subscales: Somatic Complaints, Social Isolation, Aggression, Behavior Problems, Hyperactivity/Concentration Problems and Depression/Anxiety. The item scores per subscale are summarized in a total score. The Y-OQ30 is a valid and reliable instrument for assessing change in functioning [45, 46]. The validity and reliability of the Dutch translation are currently being investigated (Baars, ongoing study).

Parents digitally completed the Kidscreen 27 and the Y-OQ30 about their child at the start of the treatment, every 3 months subsequently and finally at the end of treatment.

### Sample size

Power analysis was conducted in G*Power. Running a power analysis on a repeated measures ANOVA with four measurements, three groups, a power of 0.80, an alpha level of 0.05, and a small effect size (*f *= 0.05) [47, 48], the required total sample size was 129 for an unclustered RCT (43 patients per arm). To adjust for within-cluster correlation, we calculated the design effect or inflation factor (Design effect = 1+(*m *− 1)*ρ,* whereby *m* is the average cluster size and *ρ* is the intraclass correlation coefficient or ICC) [49, 50]. Based on pilot data, in this study, the average cluster size was set at *m* = 20 and the *ρ* = 0.001. The design effect was, therefore, set at 1.03. The total sample size for the cluster RCT was 132 patients.

### Randomization

The randomization procedure was based on random number tables and was performed using a computer-generated sequence with allocation concealment. The random allocation of the location clusters was performed before patient recruitment and enrolment started. Accordingly, all participating therapists associated with the same Autism Care Team and their patients were randomized into the same condition. A total of eight clusters were randomized among the two conditions. The average cluster size was 20.75 with a standard deviation of 10.57.

### Statistical methods

Analyses were performed according to the intention-to-treat (ITT) principle including every subject who had been randomized according to the randomized treatment assignment. Demographic variables, diagnosis characteristics of the participating patients and treatment characteristics were summarized using descriptive statistics and compared between the experimental and control group to verify prognostic comparability at baseline. Baseline characteristics are presented in mean (*M*) and Standard Deviation (SD) for continuous variables, or as frequencies and percentages for categorical variables. To check for possible differences between the three groups, we used an ANOVA for continuous variables or *X*^*2*^ test for categorical variables.

As assessed by inspection of a boxplot, we identified three participants with outlier scores due to an unusually high (more than 3 SD), but not invalid score at one measurement point: three times for the total score of the Y-OQ30, two for the subscales Aggression, and Depression and Fear and one for the subscale Conduct Problems all of the Y-OQ30. Because these were valid scores, we decided to keep them in the analysis. To examine the effect of removing the outliers, we have re-run the analyses without outliers.

Scores for each group were normally distributed, as assessed using Shapiro–Wilk’s test (*p* > 0.05), and variances were homogeneous, as assessed using Levene’s test for equality of variances for all variables.

To estimate group differences in rates of change in QoL (Kidscreen-27) or rates of change in symptom severity level (Y-OQ30), we used mixed-effects linear models with repeated measures. These models allow using all available data of subjects with randomly missing data and take into account unequal intervals between assessments, as was the case between time points 4 and 5 and also the hierarchical structure and dependency in the data. The repeated measures are correlated within participants and are nested in the two groups (FIT or CAU). Although each group was nested in clusters of four (clinic) locations (as result of the cluster randomization), the ICC was small (Kidscreen-27 total score ICC = 0.0081; Y-OQ30 total score ICC = 0.0215); therefore, location was not a contextual variable affecting the outcome, and therefore was left out of our model. The predictors in the models were Time, Group and Time × Group. All models included fixed effects for Time and Group and the interaction between Time and Group. The models also included a random effect of individual intercepts, taking into account the correlated data within individuals. The overall group comparison was followed by post hoc pairwise comparison. Correction for multiple comparisons was applied to the overall analyses using the False Discovery Rate (FDR) with an FDR adjusted *p* value setting of 0.05 [51]. For these analyses, Statistical Package for the Social Sciences (SPSS) for Windows, Version 22.0 (SPSS Inc., Chicago, Ill, USA) software was used.

## Results

### Baseline characteristics

Table 1 shows the baseline demographic and clinical characteristics of the participants. Overall, no group differences were found for age (mean CAU 10.2, SD ± 3.0; FIT 3–8 11.1, SD ± 2.9; FIT 9 + 10.9, SD ± 3.2; *p* = 0.28), gender (CAU 75.0% male; FIT 3–8 73.2% male; FIT 9 + 68.9%; *p* = 0.76) and diagnosis (CAU 78.8% ASD, 15.0% Attention-Deficit Hyperactivity Disorder (ADHD), 6.3% other; FIT 3–8 87.5% ASD, 9.8% ADHD, 2.4% other; FIT 9 + 86.6% ASD 0.0% ADHD, 13.3% other; *p* = 0.10). In the FIT 9 + group, Cognitive Behavioral Therapy (*p* < 0.001) was more frequently offered, in the CAU more Psycho-Education (*p* = 0.03) and in the FIT 3–8 slightly more Expressive/Psychomotoric Therapy (*p* = 0.43).

**Table 1:** Baseline demographic and clinical characteristics of the participants

	Care as usual (*n* = 80)		FIT 3–8 sessions (n = 41)		FIT 9 + sessions (*n* = 45)		*p* value
Age (year), (mean, ± SD)	10.2	± 3.0	11.1	± 2.9	10.9	± 3.2	0.277
Gender, *n* (%)							
Male	60	75.0%	30	73.2%	31	68.9%	0.761
Female	20	25.0%	11	26.8%	14	31.1%	
Diagnosis, *n* (%)							
Attention-deficit hyperactivity disorder	12	15.0%	4	9.8%	0	0.0%	0.103
Autistic disorder	20	25.0%	11	26.8%	10	22.2%	
Pervasive developmental disorder-NOS	34	42.5%	21	51.2%	20	44.4%	
Asperger syndrome	9	11.3%	4	9.8%	9	20.0%	
Other	5	6.3%	1	2.4%	6	13.3%	
Therapy received							
Cognitive behavioral therapy, *n* (%)	24	24.7%	18	18.6%	35	36.1%	0.000
Pharmaceutical therapy, *n* (%)	48	40.0%	20	16.7%	23	19.2%	0.423
Family and system therapy, *n* (%)	36	45.6%	16	20.3%	13	16.5%	0.208
Parental guidance, *n* (%)	46	33.3%	28	20.3%	30	21.7%	0.412
Psycho-education, *n* (%)	45	35.4%	27	21.3%	36	28.3%	0.028
Psychotherapy, *n* (%)	76	37.3%	40	19.6%	44	21.6%	0.653
Supportive treatment patient, *n* (%)	20	52.6%	9	23.7%	4	10.5%	0.089
Skills training, *n* (%)	9	27.3%	5	15.2%	10	30.3%	0.220
Expressive/psycho-motoric therapy, *n* (%)	4	13.3%	8	26.7%	6	20.0%	0.043
CBCL 6-18 *baseline*							
Anxious/depressed, (mean, ± SD)	8.4	± 5.3	7.8	± 5.2	9.2	± 5.3	0.465
Withdrawn/depressed, (mean, ± SD)	5.7	± 3.3	7.7	± 3.8	6.4	± 3.5	0.021
Somatic complaints, (mean, ± SD)	3.8	± 3.3	4.0	± 4.1	5.4	± 4.3	0.087
Social problems, (mean, ± SD)	7.2	± 3.5	6.6	± 3.6	7.5	± 4.0	0.581
Thought problems, (mean, ± SD)	7.1	± 3.9	6.5	± 4.1	7.0	± 4.0	0.752
Attention problems, (mean, ± SD)	10.8	± 3.9	9.6	± 3.9	9.6	± 3.7	0.134
Rule-breaking behavior, (mean, ± SD)	4.8	± 3.3	4.4	± 4.1	3.6	± 2.8	0.188
Aggressive behavior, (mean, ± SD)	14.1	± 7.4	13.5	± 8.3	12.9	± 7.4	0.724
Internalizing behavior, (mean, ± SD)	17.9	± 9.3	19.4	± 11.5	21.0	± 10.7	0.304
Externalizing behavior, (mean, ± SD)	18.9	± 9.7	17.9	± 11.8	16.4	± 9.4	0.486
Total score, (mean, ± SD)	69.0	± 23.7	66.0	± 30.3	68.0	± 25.3	0.845
KIDSCREEN-27 *baseline*							
Autonomy and parent relation	26.6	± 3.7	27.5	± 3.6	26.9	± 3.6	0.489
Peers and social support	13.2	± 3.4	13.0	± 3.2	13.2	± 3.3	0.954
School environment	13.9	± 3.0	12.7	± 3.2	14.0	± 2.7	0.062
Physical well-being	16.9	± 3.7	15.5	± 3.6	15.7	± 3.9	0.099
Psychological well-being	25.0	± 4.3	23.1	± 5.0	23.3	± 4,2	0.036
Total score	95.6	± 12.6	91.8	± 12.6	93.0	± 11.0	0.226
Y-OQ-30 *baseline*							
Somatic complaints	3.7	± 2.6	3.8	± 3.3	4.4	± 2.9	0.459
Social isolation	2.6	± 2.0	3.4	± 2.5	3.2	± 2.2	0.189
Aggression	2.2	± 2.6	2.5	± 3.0	2.4	± 2.3	0.858
Behavioral problems	7.4	± 4.9	7.0	± 5.4	6.5	± 4.4	0.633
Hyperactivity and concentration problems	7.4	± 2.6	6.8	± 2.9	6.8	± 3.1	0.484
Depression and fear	6.2	± 3.6	8.0	± 5.2	8.0	± 4.6	0.057
Total score	38.8	± 15.4	40.8	± 19.5	41.0	± 15.9	0.752

We ran an ANOVA to determine if there were differences at baseline for the total scores on the CBCL 6–18, the Kidscreen 27 and the Y-OQ30 between the CAU and FIT groups. Table 1 shows there was no significant difference on the CBCL total score between the CAU (*M* = 69.0, SD = 23.7), FIT 3–8 (*M* = 66.0, SD = 30.3) and the FIT 9 + (*M* = 68.0, SD = 25.3) *(p* = 0.85). However, we did find a significantly higher score on the subscale Withdrawn/Depressed in the FIT 3–8 *(p* = 0.02). There was no significant difference between the baseline total scores on the Kidscreen 27 between the CAU (*M *= 95.6, SD= 12.6), the FIT 3–8 (*M *= 91.8, SD= 12.6) and the FIT 9 + (*M *= 93.0, SD= 11.0) (*p *= 0.23). There was a significantly higher score on the subscale Psychological Well-being for the CAU (*p* = 0.04). Furthermore, no significant difference was found between the baseline total scores on the Y-OQ30 between the CAU (*M *= 38.8, SD= 15.4), the FIT 3–8 group (*M *= 40.8, SD= 19.5) and the FIT 9 + group (*M *= 41.0, SD= 15.9) (*p *= 0.75). For the subscale Depression and Fear, there was a significantly higher score for the FIT 3–8 (*p *= 0.57). After removing the outliers from the analyses, there were no significant differences for this subscale.

### Effect of treatment: primary outcome QoL

**Fig. 2:**
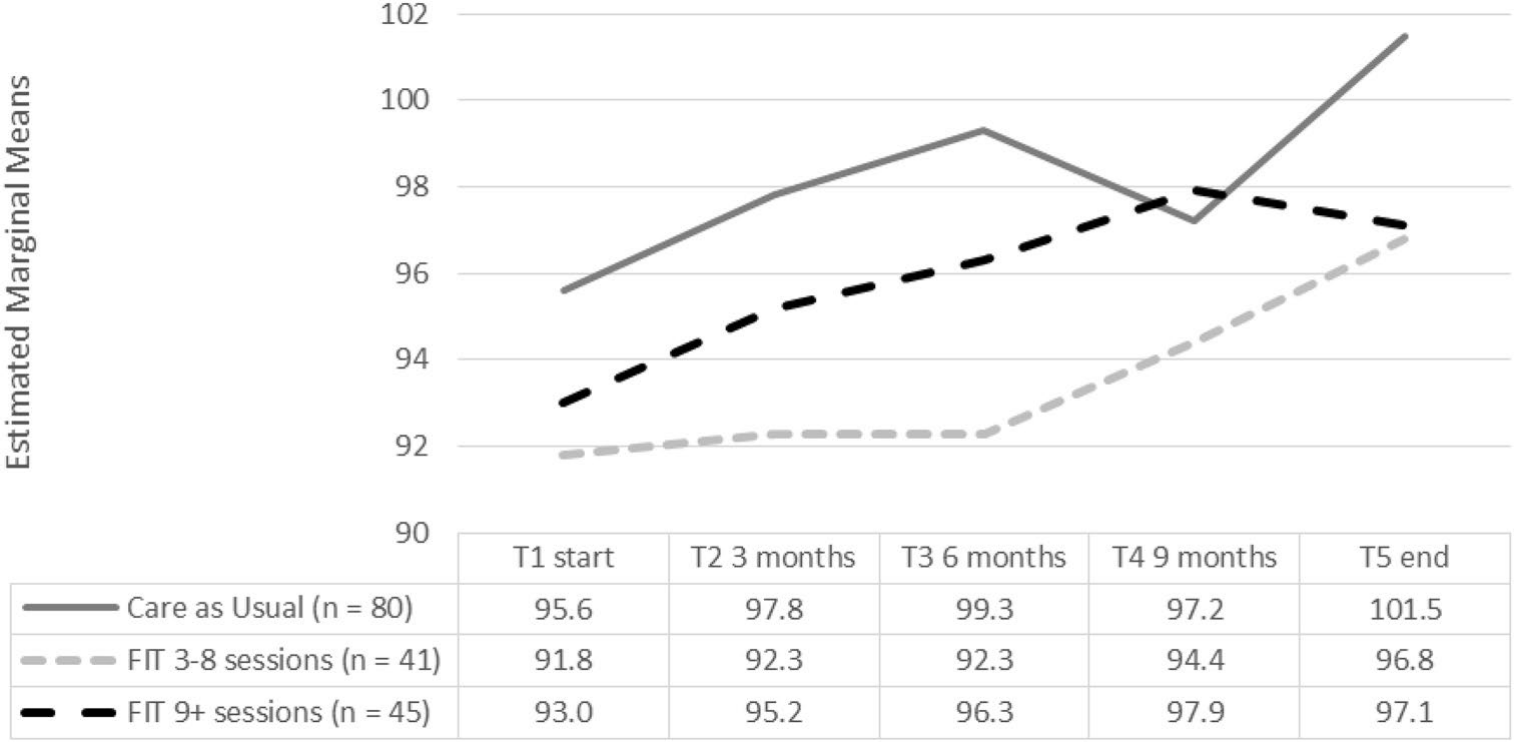
Estimated marginal means of the total score on the Kidscreen-27 at T1, T2, T3, T4 and T5

As shown in Table 2 and Fig. 2, we conducted linear mixed modeling to examine Time by Group interactions for the primary and secondary outcome measures. For the QoL total score, we found a significant effect for Time [*F* (4, 465) = 7.92, *p* < 0.00] and for Group [*F* (2, 165) = 3.16, *p* = 0.045], but no interaction effect was found [*F* (8, 465) = 0.96 *p* = 0.47]. Treatment over time had a positive influence on improvement in QoL, as well as adding FIT to treatment. The effects of Time and Group did not reinforce each other. Post hoc pairwise comparisons showed that this difference for Group was seen between the FIT 3–8 and CAU.

**Table 2:** Scores KIDSCREEN-27 at T1 start, T2, T3, T4 and T5

	Care as usual			FIT 3–8 sessions			FIT 9 + sessions				Linear mixed-effects with repeated measures										
	(*n* = 80)			(*n* = 41)			(*n* = 45)				TIME				GROUP				Interaction TxG^F^		
	*N*	Mean	±SD	*N*	Mean	±SD	*N*	Mean	±SD		Post hoc comparison	*df*_1_, *df*_2_	*F*	*p* value	Post hoc comparison	*df*_1_, *df*_2_	*F*	*p* value	*df*_1_, *df*_2_	*F*	*p* value
Autonomy and parent relation																					
Start	80	26.6	± 3.7	41	27.5	± 3.6	45	26.9	± 3.6		Start (ref.)	4, 462	5.20	0.000	CAU (ref.)	2, 160	0.78	0.461	8, 462	1.72	0.092
3 months	46	27.5	± 4.0	30	27.5	± 3.5	34	27.7	± 3.4		3 months				3–8 sessions						
6 months	43	28.1	± 3.3	31	27.3	± 2.8	38	27.8	± 2.9		6 months				9 + sessions						
9 months	46	27.1	± 3.4	27	27.5	± 2.1	33	28.9	± 3.1		9 months *										
End	52	28.4	± 3.2	32	27.8	± 3.0	40	28.3	± 3.1		End ***										
Peers and social support																					
Start	80	13.2	± 3.4	41	13.0	± 3.2	45	13.2	± 3.3		Start (ref.)	4, 476	2.34	0.054	CAU (ref.)	2, 168	2.20	0.114	8, 476	1.38	0.203
3 months	46	13.9	± 3.2	30	12.2	± 3.2	34	12.9	± 2.7		3 months				3–8 sessions						
6 months	43	14.2	± 2.9	31	12.0	± 2.7	38	13.2	± 3.1		6 months				9 + sessions						
9 months	46	13.4	± 3.4	27	13.3	± 2.6	33	13.0	± 3.7		9 months										
End	52	14.4	± 3.7	32	13.9	± 2.1	40	13.4	± 3.9		End										
School environment																					
Start	80	13.9	± 3.0	41	12.7	± 3.2	45	14.0	± 2.7		Start (ref.)	4, 476	2.39	0.050	CAU (ref.)	2, 163	3.93	0.022	8, 477	1.00	0.267
3 months	46	14.6	± 3.0	30	13.3	± 3.3	34	13.5	± 3.1		3 months				3–8 sessions *						
6 months	43	15.3	± 2.4	31	13.0	± 3.7	38	14.7	± 2.6		6 months				9 + sessions						
9 months	46	14.7	± 3.3	27	13.7	± 2.8	33	14.0	± 3.1		9 months										
End	52	14.5	± 3.5	32	14.6	± 2.5	40	14.0	± 3.7		End *										
Physical well-being																					
Start	80	16.9	± 3.8	41	15.5	± 3.6	45	15.7	± 3.9		Start (ref.)	4, 463	1.08	0.366	CAU (ref.)	2, 166	4.00	0.020	8, 463	1.20	0.295
3 months	46	16.3	± 3.9	30	15.3	± 3.4	34	15.5	± 3.0		3 months				3–8 sessions*						
6 months	43	17.4	± 3.9	31	15.7	± 3.4	38	16.2	± 3.8		6 months				9 + sessions						
9 months	46	17.0	± 3.9	27	14.9	± 2.8	33	16.3	± 3.1		9 months										
End	52	17.4	± 3.7	32	16.0	± 3.1	40	15.3	± 3.3		End										
Psychological well-being																					
Start	80	25.0	± 4.3	41	23.1	± 5.0	45	23.3	± 4.2		Start (ref.)	4, 465	10.74	0.000	CAU (ref.)	2, 164	1.93	0.148	8, 466	1.01	0.430
3 months	46	26.0	± 4.1	30	24.3	± 4.8	34	24.2	± 4.4		3 months				3–8 sessions						
6 months	43	25.7	± 4.2	31	24.6	± 4.2	38	24.9	± 4.4		6 months*				9 + sessions						
9 months	46	25.3	± 3.5	27	24.4	± 4.0	33	25.8	± 4.0		9 months *										
End	52	26.4	± 3.7	32	25.8	± 3.7	40	25.7	± 3.7		End ***										
Total score																					
Start	80	95.6	± 12.6	41	91.8	± 12.6	45	93.0	± 11.0		Start (ref.)	4, 465	7.92	0.000	CAU (ref.)	2, 165	3.16	0.045	8, 465	0.96	0.469
3 months	46	98.3	± 12.8	30	92.7	± 12.0	34	93.7	± 10.5		3 months				3–8 sessions *						
6 months	43	100.7	± 11.7	31	92.6	± 11.3	38	96.8	± 10.9		6 months				9 + sessions						
9 months	46	97.5	± 13.3	27	93.8	± 10.3	33	98.0	± 11.8		9 months *										
End	52	101.1	± 12.5	32	98.0	± 9.1	40	96.7	± 11.4		End ***										

Results linear mixed-effects analysis

*T* main effect for time, *G* main effect for group, *TxG* time × group interaction, *F* False Discovery Rate correction for multiple comparisons; *KIDSCREEN-27* higher scores means higher quality of life

*p < 0.05; **p < 0.005; ***p < 0.0001

A positive and significant effect was found for Group on the subscale School Environment [*F* (2,163) = 3.93, *p *= 0.02] and Physical Well-being [*F* (2,166) = 4.00, *p *= 0.02]. Post hoc pairwise comparison showed that this difference was between CAU and 3–8 FIT. However, no interaction effect was found for both the subscale Physical Well-being and School Environment.

Significant positive effects for Time on subscale level were found for Autonomy and Parent Relation [*F* (4,462) = 5.2 *p *< 0.00], School Environment [*F* (4,476) = 2.39 *p *=0.05] and Psychological Well-being [*F* (4,465) = 10.74 *p *< 0.00].

### Effect of treatment: secondary outcome Symptom Severity

**Fig. 3:**
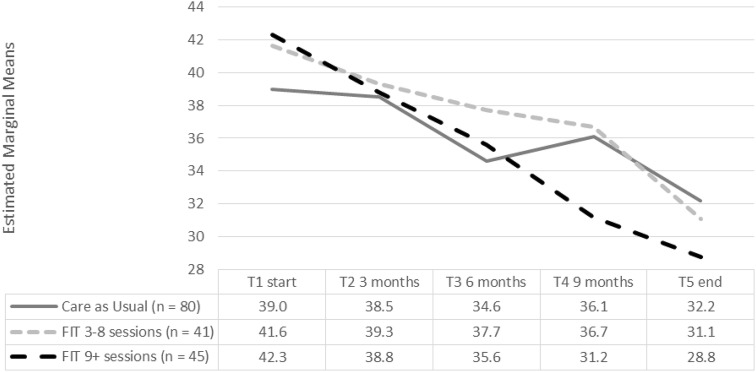
Estimated marginal means of the total score on the Y-OQ-30 at T1, T2, T3, T4 and T5

For the Symptom Severity outcome (Y-OQ30), as shown in Table 3 and Fig. 3, we found a significant effect for Time [*F* (4, 409) = 23.89, *p* < 0.001], but not for Group [*F* (2, 158) = 0.19, *p *= 0.83] and there was no interaction effect for Time by Group [*F* (8, 409) = 1.72, *p* = 0.09]. All participants profit from treatment, but the addition of FIT did not create a significant difference between the groups in symptom severity reduction.

**Table 3:** Scores Y-OQ-30 at T1 start, T2, T3, T4 and T5

	Care as usual			FIT 3–8 sessions			FIT 9 + sessions				Linear mixed-effects with repeated measures										
	(*n* = 80)			(*n* = 41)			(*n* = 45)				TIME				GROUP				Interaction TxG^F^		
	*N*	Mean	±SD	*N*	Mean	±SD	*N*	Mean	±SD		Post hoc comparison	*df*_1_, *df*_2_	*F*	*p* value	Post hoc comparison	*df*_1_, *df*_2_	*F*	*p* value	*df*_1_, *df*_2_	*F*	*p* value
Somatic complaints																					
Start	71	3.6	± 2.6	31	3.9	± 3.3	37	4.6	± 2.9		Start (ref.)	4, 413	6.55	0.000	CAU (ref.)	2, 159	1.69	0.188	8, 413	0.53	0.835
3 months	43	3.1	± 2.8	27	3.8	± 2.7	32	4.8	± 3.0		3 months				3–8 sessions						
6 months	43	3.1	± 2.6	29	3.4	± 2.5	33	3.9	± 2.9		6 months *				9 + sessions						
9 months	47	2.8	± 2.5	24	3.9	± 2.8	27	3.6	± 2.4		9 months										
End	53	3.0	± 3.1	30	3.3	± 2.6	36	3.6	± 2.8		End ***										
Social isolation																					
Start	71	2.6	± 2.0	31	3.4	± 2.5	37	3.3	± 2.2		Start (ref.)	4, 413	5.17	0.000	CAU (ref.)	2, 156	2.70	0.070	8, 414	1.29	0.247
3 months	43	2.8	± 2.1	27	3.9	± 2.4	32	3.1	± 1.8		3 months				3–8 sessions						
6 months	43	2.6	± 2.2	29	3.8	± 1.7	33	2.6	± 2.1		6 months				9 + sessions						
9 months	47	2.7	± 2.0	24	2.9	± 1.9	27	2.4	± 2.2		9 months										
End	53	2.3	± 2.0	30	3.2	± 2.2	36	2.1	± 1.7		End **										
Aggression																					
Start	71	2.2	± 2.6	31	2.4	± 3.0	37	2.5	± 2.3		Start (ref.)	4, 412	8.47	0.000	CAU (ref.)	2, 159	0.56	0.575	8, 413	1.37	0.205
3 months	43	2.1	± 2.5	27	2.1	± 3.1	32	1.5	± 1.7		3 months				3–8 sessions						
6 months	43	2.1	± 2.5	29	1.9	± 2.4	33	1.6	± 1.9		6 months				9 + sessions						
9 months	47	1.9	± 2.4	24	1.6	± 2.7	27	1.4	± 1.7		9 months										
End	53	1.8	± 2.5	30	0.9	± 1.3	36	1.3	± 1.6		End ***										
Behavioral problems																					
Start	71	7.4	± 5.0	31	6.7	± 5.3	37	6.8	± 4.3		Start (ref.)	4, 410	13.00	0.000	CAU (ref.)	2, 160	1.65	0.195	8, 410	0.96	0.463
3 months	43	6.7	± 4.6	27	6.6	± 6.2	32	5.9	± 3.4		3 months				3–8 sessions						
6 months	43	6.1	± 4.7	29	6.8	± 4.8	33	5.4	± 3.4		6 months *				9 + sessions						
9 months	47	6.6	± 4.8	24	5.5	± 4.6	27	4.3	± 3.2		9 months **										
End	53	5.9	± 4.5	30	4.7	± 3.9	36	4.3	± 3.1		End ***										
Hyperactivity and concentration problems																					
Start	71	7.3	± 2.6	31	6.8	± 3.0	37	6.9	± 3.2		Start (ref.)	4, 415	18.03	0.000	CAU (ref.)	2, 163	2.41	0.093	8, 416	1.97	0.049
3 months	43	7.1	± 2.9	27	6.1	± 2.6	32	6.7	± 2.9		3 months				3–8 sessions						
6 months	43	6.4	± 2.7	29	6.2	± 2.6	33	6.2	± 2.6		6 months ***				9 + sessions						
9 months	47	6.7	± 2.3	24	5.8	± 2.5	27	4.9	± 2.9		9 months ***										
End	53	6.7	± 2.6	30	5.3	± 2.9	36	5.2	± 2.6		End ***										
Depression and fear																					
Start	71	6.2	± 3.5	31	8.1	± 5.3	37	8.1	± 4.7		Start (ref.)	4, 413	11.50	0.000	CAU (ref.)	2, 160	3.55	0.031	8, 413	1.93	0.054
3 months	43	5.7	± 3.6	27	7.3	± 5.3	32	8.7	± 5.0		3 months				3–8 sessions*						
6 months	43	5.5	± 3.6	29	7.8	± 4.9	33	7.2	± 4.4		6 months				9 + sessions *						
9 months	47	6.1	± 3.1	24	7.7	± 5.0	27	6.4	± 3.7		9 months										
End	53	5.3	± 3.5	30	5.8	± 3.9	36	5.9	± 3.8		End ***										
Total score																					
Start	71	38.4	± 15.6	31	40.5	± 19.8	37	42.3	± 15.3		Start (ref.)	4, 409	23.89	0.000	CAU (ref.)	2, 158	0.19	0.825	8, 409	1.71	0.093
3 months	43	36.0	± 16.5	27	38.6	± 21.6	32	39.3	± 15.4		3 months				3–8 sessions						
6 months	43	34.0	± 16.2	29	38.3	± 15.8	33	34.8	± 15.3		6 months ***				9 + sessions						
9 months	47	34.4	± 13.6	24	36.3	± 18.8	27	30.4	± 14.0		9 months ***										
End	53	32.9	± 13.8	30	30.6	± 14.0	36	29.4	± 13.5		End ***										

Results linear mixed-effects analysis

*T* main effect for time, *G* main effect for group, *TxG* time × group interaction, *F* False Discovery Rate correction for multiple comparisons, *Y-OQ-30* higher scores means more problems

*p < 0.05; **p < 0.005; ***p < 0.0001

We did find an interaction effect for symptom severity reduction for the subscale Depression and Fear [*F* (8, 413) = 1.93 *p* = 0.05]. However, this difference may have been caused by a higher baseline score for participants in both FIT groups. This was also shown in Table 1, where we found significantly higher scores on the CBCL and the Y-OQ30 on subscales for Depression at baseline for both FIT groups. Additionally, an interaction effect for the subscale Hyperactivity and Concentrations Problems was found [*F* (8, 416) = 1.97, *p* = 0.05] suggesting that the two groups which received FIT showed a significantly higher decrease compared with the control group.

We found significant positive effects for Group for the subscale Depression and Fear [*F* (2, 160) = 3.55 *p* = 0.03]. Furthermore, we found positive effects for Time for all subscales: Somatic Complaints [*F* (4, 413) = 6.55 *p* < 0.001], Social Isolation [*F* (4, 413) = 5.17 *p* < 0.001], Aggression [*F* (4, 412) = 8.47 *p* < 0.001], Behavior Problems [*F* (4, 410) = 12.99, *p* < 0.001], Hyperactivity and Concentration Problems [*F* (4, 415) = 18.03, *p* < 0.001], Depression and Fear [*F* (4, 413) = 11.5, *p* < 0.001].

### Sensitivity analysis

To test the robustness of the findings, we performed several separate analyses. First, we looked at the results after removing the outliers. We found a significant interaction effect for Time by Group [*F* (8, 402) = 2.10 *p* = 0.035] for the total scale of the Y-OQ30. The estimated means show that this effect applies particularly to the FIT 9 + . We also found a significant interaction effect on the subscale Depression and Fear [*F* (8, 407) = 2.23, *p* = 0.024] and a significant interaction effect for the subscale Hyperactivity and Concentration problems [*F* (8, 409) = 1.99, *p* = 0.05].

Secondly, we examined the effect of Time by Severity interactions for the KIDSCREEN-27 total score and the Y-OQ30 total score. To define Severity, we used the clinical cut-off scores of the CBCL at baseline (normal, borderline and clinical). As for the KIDSCREEN-27, we found a significant effect for Severity [*F* (2, 147) = 7.908, *p* = 0.001]. The estimated marginal means of the KIDSCREEN-27 for children with clinical, borderline and normal scores were 94.2, 100.0 and 102.1, respectively. Post hoc pairwise comparisons showed that this difference in QoL was seen in children with a score in the normal range versus children with clinical scores. No interaction effect was found for Severity by Time [*F* (8, 422) = 1.525 *p* = 0.147].

Examining the Y-OQ30, a significant effect for Severity [*F* (2, 143) = 23.6, *p* < 0.000] was found. The estimated marginal means of the Y-OQ30 for clinical, borderline and normal scores were 40.2, 25.9 and 22.4, respectively. No interaction effect was found for Severity by Time [*F* (8, 374) = 0.452 *p* = 0.889]. These results suggest that independent of Severity, all groups showed a decrease in symptom severity outcome over Time, but the decreases in scores were equal in all three groups.

Furthermore, we performed a subgroup analysis, selecting children who scored clinically on the CBCL at baseline. No significant Time × Group interactions were seen for both outcome variables.

To test the robustness of the findings with respect to similarities within the clusters, we also performed a sensitivity analysis, considering the clusters. For the outcomes, we defined the following levels: the repeated observations (level 1) nested within Group (level 2) nested within Location (level 3). No interaction effect was found for the KIDSCREEN-27 total score [*F* (47, 475) = 1.028, *p* < 0.426], nor for the Y-OQ30 total score [*F* (55, 404) = 1.231, *p* < 0.136].

## Discussion

The purpose of this study was to investigate the efficacy of adding FIT to CAU in a child and adolescent psychiatry setting. The main finding of this study is that the systematic use of patients’ feedback in treatment leads to a more pronounced increase of QoL. Although adding FIT to treatment is effective in gaining a more significant increase in QoL, no additional effects were seen in decreasing symptom severity. Interestingly, no interaction with the duration of treatment was found.

The positive effect of FIT on QoL is certainly interesting because in previous feedback studies treatment outcome has rarely been measured in terms of QoL [23]. This is remarkable because both QoL and patients’ feedback emphasize the patients’ view of their situation. It has been stated that QoL is the subjective perception and evaluation by the patient of their situation [27]. In our study, the QoL of the children was reported by the parents and therefore can be viewed as a measurement of the abilities and functioning of the child by the parent [27, 52]. We hypothesize that the parents’ conceptualization of the child’s abilities depends on their level of distress based on their judgement of the severity of the ASD. Their distress also depends on the extent to which others, like therapists, meet their needs. In FIT, the emphasis is on therapists fostering supportive interactions with parents and the child and monitoring if they are meeting their needs. Positively influencing patients’ expectations, as is incorporated in FIT, is known as an important factor in treatment [8]. By doing so, therapists using FIT can enhance a positive view of the child’s QoL as seen through the eyes of the parent. The effect of FIT on patients’ expectations is an interesting topic for further research. It could be speculated that only the patients can report the QoL as a subjective concept (e.g., the child or adolescent) [27, 52]. Conceptualized in this way, it would be very interesting to study QoL measures between the child or adolescent, and their parents [27].

Adding FIT had no significant effect on decreasing symptom severity, although, based on the literature, we had expected a positive effect [1–4]. Our findings, however, are in line with the meta-review of the Cochrane Collaboration [23]. It is important to note that, to the best of our knowledge, no feedback study has been reported with children with ASD. It could be considered that the symptom severity for children with ASD is thought to be a ‘life-long’ condition, which is partially supported by data on adults with autism [53], at least with respect to symptom distributions in different age cohorts. At the same time, we found a significant decrease in symptom severity in our ASD sample over time, although this should be carefully interpreted since we did not specifically measure the core symptoms of ASD. Current research on the stability of ASD symptom severity over time has concluded that changes do indeed occur in developmental trajectories of ASD, in both directions [54, 55]. Earlier research on moderating factors suggests that the positive effects of using feedback are stronger for patients who are at risk of treatment failure or who deteriorate during treatment (known as ‘patients not on track (NOT) to reach their goal for therapy’) [1, 3–6]. In our sample, we were unable to study the effects of such moderating factors, partly because yet there are no reliable Dutch norms for the ORS to define when a patient is not on track.

In this study, FIT was added to care as usual. The therapists using FIT were asked to discuss the feedback outcome as shown in the graph during treatment. We did not measure whether the feedback was used to tailor the general treatment plan as prescribed in the care as usual, nor in what way the therapists changed their attitude or behavior. It is suggested that a more deliberate practice by the therapist, based on feedback is a moderator for a positive effect of feedback on treatment outcome [56, 57]. The effect of feedback on outcome may also be moderated by therapist characteristics, in particular, their attitude and openness to the patient’s feedback [18, 19]. In our study, we were not able to analyze the data regarding this issue. In addition, these factors might imply a moderating role of the organizational aspects, social context, etc. [12, 13]. It is beyond the scope of this current study to take all these into account, but it is highly recommended for further research to sort out the effects of such moderators systematically.

We suspect that specific child characteristics may also moderate the effect of feedback. Duncan et al. concluded that the child’s self-reported ORS is positively correlated with the caregiver’s view and is, therefore, a reliable and valid marker of treatment progress [37]. Our sample was a group with ASD. We chose this group because it is the largest patient group in our treatment center (*Karakter*). Therapists in this study mentioned that some children had difficulties in completing the ORS or SRS in a sensible way. The FIT supervisor emphasized the value of talking about how the children experience the treatment even if these children had difficulties in completing the questionnaires. It is possible that FIT does not function optimally under these conditions. We recommend extending this research to other child psychiatric samples with severe problems, including ADHD, Depression or Anxiety disorders, Behavioral problems or Personality disorders.

This study showed a significant increase in QoL and a significant decrease in symptom severity by following treatment over a certain period of time, which positively underscores the importance of treatment availability for children. The effect of FIT on QoL was found for the group with 3–8 FIT sessions. Although we expected a positive relationship between the frequency of feedback and outcome [4, 31], we did not find it in our study. The different effect between the two FIT groups cannot be explained by differences in room for improvement depending on symptom severity, since the groups did not differ in severity at baseline and, in addition, we found no interaction effect for severity. As mentioned before, we did not find a robust interaction effect between Time and adding FIT. We expected a stronger effect for FIT, mainly in the first episode. This would be in line with previous findings about trajectories of change, with most change occurring earlier rather than later in the treatment process indicating, the importance of the critical initial phase (see Miller et al. [58] and also Amble et al. [59] for an overview). Our finding suggests we should reconsider the Time factor. Changes in treatment do not occur in a linear way but appear with sudden gains and sudden losses at different time moments. A broader and very intriguing reflection on this finding is found by Schiepek et al. [60, 61]. Based on Synergetic theories, they examined treatment as a complex, dynamic, non-linear process of change. They emphasize the importance of the use of patients’ feedback in creating sensitivity within the therapist and the patient for critical stages in treatment as a change process [62]. We are extending our theoretical feedback model for further research to take this into account [63].

### Limitations

The results of this study should be interpreted in the context of certain limitations. Although we are convinced of the importance of performing treatment effect research in a realistic clinical environment, we had to accept some practical limitations. Due to organizational context factors, such as a considerable time delay between the diagnostics phase and the treatment phase, we extended the inclusion by 6 months and then terminated for budgetary reasons. During the inclusion and treatment phase, several therapists changed teams due to organizational reorganization. Although these changes did not influence the comparability of the therapist groups, we had to make a stronger effort to keep adherence to the protocol. Due to organizational and administrative reasons, we were limited in calculating the ratio between the number of FIT sessions and the total number of treatment sessions. Interestingly, we noticed that most of the therapists showed enthusiasm for using FIT as an important communication tool with patients. Most of the FIT teams decided to continue using FIT after the study ended.

Earlier, we mentioned a limitation of not being able to define and administer when a patient is NOT. It is recommended to develop norms to be able to adequately define and assess NOT for further research.

Another limitation was the relatively small sample size as indicated by the rather strong effect of the outlier scores. Our second analysis, without the outlier scores for the Y-OQ30, showed a significant interaction effect for Time and Group on symptom severity, where keeping the outlier scores in the analysis decreases the significance to non-significant. A larger sample size would have minimized the effect of a possible outlier.

We hypothesize another possible limitation: we could not control for a conceivably natural tendency of therapists in the control group to be attentive to what is important for the patient. The effect of the systematic use of feedback in the FIT group on treatment outcome in comparison with the CAU group could be somewhat weakened by these natural phenomena in the CAU group which fits in the democratizing process of healthcare.

## Conclusion

In conclusion, the systematic use of FIT in a Child and Adolescent Psychiatric setting may increase QoL but does not seem to result in a more pronounced decrease in symptom severity compared with CAU. It is suggested that FIT changes the expectations of parents in a positive way. These results should be replicated in other samples and include an extensive study on the moderating factors that may be involved.

## References

[1] Gondek D, Edbrooke-Childs J, Fink E (2016). Feedback from outcome measures and treatment effectiveness, treatment efficiency, and collaborative practice: a systematic review. Adm Policy Ment Heal Ment Heal Serv Res.

[2] Knaup C, Koesters M, Schoefer D (2009). Effect of feedback of treatment outcome in specialist mental healthcare: meta-analysis. Br J Psychiatry.

[3] Carlier IVE, Meuldijk D, Van Vliet IM (2012). Routine outcome monitoring and feedback on physical or mental health status: evidence and theory. J Eval Clin Pract.

[4] Lambert MJ, Shimokawa K (2011). Collecting client feedback. Psychotherapy.

[5] Lutz W, De Jong K, Rubel J (2015). Patient-focused and feedback research in psychotherapy: where are we and where do we want to go?. Psychother Res.

[6] Shimokawa K, Lambert MJ, Smart DW (2010). Enhancing treatment outcome of patients at risk of treatment failure: meta-analytic and mega-analytic review of a psychotherapy quality assurance system. J Consult Clin Psychol.

[7] Lambert M (2007). Presidential address: what we have learned from a decade of research aimed at improving psychotherapy outcome in routine care. Psychother Res.

[8] Wampold BE (2015). How important are the common factors in psychotherapy? An update. World Psychiatry.

[9] Ardito RB, Rabellino D (2011). Therapeutic alliance and outcome of psychotherapy: historical excursus, measurements, and prospects for research. Front Psychol.

[10] Norcross JC, Duncan Barry L, Miller Scott D, Wampold Bruce E, Hubble MA (2010). The therapeutic relationship. The heart and soul of change: delivering what works in therapy.

[11] Zack SE, Castonguay LG, Boswell JF (2007). Youth working alliance: a core clinical construct in need of empirical maturity. Harv Rev Psychiatry.

[12] Glisson C (2002). The organizational context of children’s mental health services. Clin Child Fam Psychol Rev.

[13] Tang PC, Smith MD (2016). Democratization of health care. J Am Med Assoc.

[14] Hafkenscheid A (2015). Tegenoverdracht: van een psycho- analytisch naar een transtheoretisch concept. Tijdschr Psychiatr.

[15] Prescott DS, Maeschalck CL, Miller SD (2017). Feedback-informed treatment in clinical practice: reaching for excellence.

[16] Krägeloh CU, Czuba KJ, Billington DR (2015). Using feedback from patient-reported outcome measures in mental health services: a scoping study and typology. Psychiatr Serv.

[17] Davidson K, Perry A, Bell L (2015). Would continuous feedback of patient’s clinical outcomes to practitioners improve NHS psychological therapy services? Critical analysis and assessment of quality of existing studies. Psychol Psychother Theory Res Pract.

[18] Lutz W, Rubel J, Schiefele AK (2015). Feedback and therapist effects in the context of treatment outcome and treatment length. Psychother Res.

[19] de Jong K, van Sluis P, Nugter MA (2012). Understanding the differential impact of outcome monitoring: therapist variables that moderate feedback effects in a randomized clinical trial. Psychother Res.

[20] Hawkins EJ, Lambert MJ, Vermeersch DA (2004). The therapeutic effects of providing patient progress information to therapists and patients. Psychother Res.

[21] Probst T, Lambert MJ, Loew TH (2013). Feedback on patient progress and clinical support tools for therapists: improved outcome for patients at risk of treatment failure in psychosomatic in-patient therapy under the conditions of routine practice. J Psychosom Res.

[22] Lambert MJ, Harmon C, Slade K (2005). Providing feedback to psychotherapists on their patients’ progress: clinical results and practice sugges tions. J Clin Psychol.

[23] Kendrick T, Stuart B, Gilbody S (2016). Routine use of patient reported outcome measures (PROMs) for improving treatment of common mental health disorders in adults (Review). Cochrane Databse Syst Rev.

[24] Bickman L, Kelley SD, Breda C (2011). Effects of routine feedback to clinicians on mental health outcomes of youths: results of a randomized trial. Psychiatr Serv.

[25] Stein BD, Kogan JN, Hutchison SL (2010). Use of outcomes information in child mental health treatment: results from a pilot study. Psychiatr Serv.

[26] Bullinger M (2002). Assessing health related quality of life in medicine. An overview over concepts, methods and applications in international research. Restor Neurol Neurosci.

[27] Ravens-Sieberer U, Erhart M, Wille N (2006). Generic health-related quality-of-life assessment in children and adolescents: methodological considerations. Pharmacoeconomics.

[28] Priebe S, McCabe R, Bullenkamp J (2007). Structured patient clinician communication and 1-year outcome in community mental healthcare: cluster randomised controlled trial. Br J Psychiatry.

[29] Slade M, McCronde P, Kuipers E (2006). Use of standardised outcome measures in adult mental health services: randomised controlled trial. Br J Psychiatry.

[30] American Psychiatric Association (2000). Diagnostic and statistical manual for mental disorders (4th edn. text revision) (DSM-IV-TR).

[31] Sapyta J, Riemer M, Bickman L (2005). Feedback to clinicians: theory, research, and practice. J Clin Psychol.

[32] Bargman S, Robinson B (2012). The ICCE manuals on feedback-informed treatment (fit). manual 2: feedback-informed clinical work: the basics.

[33] Thorn BE (2007). Evidence-based practice in psychology. J Clin Psychol.

[34] Tilsen J, Mcnamee S (2015). Feedback informed treatment: evidence-based practice meets social construction. Fam Process.

[35] Hafkenscheid A, Duncan BL, Miller SD (2010). The outcome and session rating scales: a cross-cultural examination of the psychometric properties of the dutch translation. J Br Ther.

[36] Janse P, Boezen-Hilberdink L, van Dijk MK (2014). Measuring feedback from clients. Eur J Psychol Assess.

[37] Duncan BL, Sparks JA, Miller SD (2006). Giving youth a voice: a preliminary study of the reliability and validity of a brief outcome measure for children, adolescents, and caretakers. J Br Ther.

[38] Achenbach TM, Rescorla LA (2001). Manual for the ASEBA school-age forms & profiles.

[39] Verhulst FC, van der Ende J, Koot HM (1996). Handleiding voor de CBCL/4-18.

[40] Ravens-Sieberer U, the European Kidscreen group (2006). The Kidscreen questionnaires: quality of life questionnaires for children and adolescents.

[41] Ravens-Sieberer U, Auquier P, Erhart M (2007). The KIDSCREEN-27 quality of life measure for children and adolescents: psychometric results from a cross-cultural survey in 13 European countries. Res Qual Life Res.

[42] Ravens-Sieberer U, Herdman M, Devine J (2014). The European KIDSCREEN approach to measure quality of life and well-being in children: development, current application, and future advances. Qual Life Res.

[43] de Jong K (2014) Outcome monitoring feedback research: Y-OQ-30. http://kimdejong.net/y-oq-30/. Accessed 17 Aug 2017

[44] Burlingame GM, Dunn TW, Hill M (2004). Administration and scoring manual for the Y-OQtm-30.2.0.

[45] Dunn TW, Burlingame GM, Walbridge M (2005). Outcome assessment for children and adolescents: psychometric validation of the youth outcome questionnaire 30.1 (Y-OQ^®^-30.1). Clin Psychol Psychother.

[46] Bybee TS, Lambert MJ, Eggett D (2007). Curves of expected recovery and their predictive validity for identifying treatment failure. Tijdschr voor Psychother.

[47] Faul F, Erdfelder E, Lang AG, Buchner A (2007). G* Power 3: a flexible statistical power analysis program for the social, behavioral, and biomedical sciences. Behav Res Methods.

[48] Faul F, Erdfelder E, Buchner A, Lang A-G (2009). Statistical power analyses using G*Power 3.1: tests for correlation and regression analyses. Behav Res Methods.

[49] Heino M (2015) Taking back the power (in cluster randomization). https://mattiheino.com/2015/10/10/taking-back-the-power-in-cluster-randomization/. Accessed 28 Nov 2017

[50] Eldridge S, Kerry S (2012). A practical guide to cluster randomised trials in health services research.

[51] Benjamin Y, Hochberg Y (2009). Controlling the false discovery rate : a practical and powerful approach to multiple testing. J Roy Stat Soc.

[52] Coghill DR, Banaschewski T, Soutullo C (2017). Systematic review of quality of life and functional outcomes in randomized placebo-controlled studies of medications for attention-deficit/hyperactivity disorder. Eur Child Adolesc Psychiatry.

[53] Siebes R, Muntjewerff JW, Staal W (2018). Differences of symptom distribution across adult age in high functioning individuals on the autism spectrum using subscales of the autism spectrum quotient. J Autism Dev Disord.

[54] Louwerse A, Eussen MLJM, Van der Ende J (2015). ASD symptom severity in adolescence of individuals diagnosed with PDD-NOS in childhood: stability and the relation with psychiatric comorbidity and societal participation. J Autism Dev Disord.

[55] Verheij C, Louwerse A, van der Ende J (2015). The stability of comorbid psychiatric disorders: a 7 year follow up of children with pervasive developmental disorder-not otherwise specified. J Autism Dev Disord.

[56] Chow DL, Miller SD, Seidel JA (2015). The role of deliberate practice in the development of highly effective psychotherapists. Psychotherapy.

[57] Miller SD, Hubble MA, Chow D, Seidel J (2015). Beyond measures and monitoring: realizing the potential of feedback-informed treatment. Psychotherapy.

[58] Miller SD, Duncan BL, Brown J (2006). Using formal client feedback to improve retention and outcome: making ongoing, real-time assessment feasible. J Br Ther.

[59] Amble I, Gude T, Ulvenes P (2016). How and when feedback works in psychotherapy: is it the signal?. Psychother Res.

[60] Schiepek G, Tominschek I, Heinzel S (2014). Self-organization in psychotherapy testing the synergetic model of change processes. Front Psychol.

[61] Heinzel S, Tominschek I, Schiepek G (2014). Dynamic patterns in psychotherapy—discontinuous changes and critical instabilities during the treatment of obsessive compulsive disorder. Nonlinear Dyn Psychol Life Sci.

[62] Schiepek G, Eckert H, Aas B (2015). Integrative psychotherapy: a feedback-driven dynamic systems approach.

[63] Salvatore S, Tschacher W, Gelo OCG, Koch SC (2015). Editorial: dynamic systems theory and embodiment in psychotherapy research. A new look at process and outcome. Front Psychol.

